# Lycium Barbarum Polysaccharides Improve Retinopathy in Diabetic Sprague-Dawley Rats

**DOI:** 10.1155/2018/7943212

**Published:** 2018-11-15

**Authors:** Qing Yao, Yi Yang, Xiaohong Lu, Qian Zhang, Mingxiu Luo, P. Andy Li, Yan Pan

**Affiliations:** ^1^Department of Biochemistry and Molecular Biology, Ningxia Medical University, Yinchuan, China; ^2^Department of Pharmaceutical Sciences, Biomanufacturing Research Institute and Technological Enterprise (BRITE), North Carolina Central University, Durham, North Carolina, USA; ^3^Department of Pharmacology, School of Basic Medical Science, Peking University, Beijing 100191, China

## Abstract

Diabetic retinopathy (DR) has become the most frequent cause of impaired visual acuity and blindness in working-age population in developed countries. Here we use diabetic rats to clarify the role of Lycium barbarum polysaccharides (LBP) on DR. We treated diabetic rats with LBP (400 mg/kg/d or 200 mg/kg/d) orally for 20 weeks. Electroretinogram (ERGs) and Laser Doppler blood flow were measured to assess the retinal function, routine histology and ultrastructural studies were performed to evaluate the morphological alterations, and immunohistochemistry, western blotting, and RT-PCR were conducted to detect the protein and mRNA levels of pro- and antiangiogenic factors. The results showed that diabetes suppressed the amplitudes of a-wave, b-wave, and oscillatory potential in ERG, reduced retinal blood flow, decreased the thickness of the retina, and increased the thickness of basement membrane of the retinal capillary. Furthermore, diabetes increased the mRNA and protein expressions of proangiogenic GFAP and VEGF and suppressed the levels of antiangiogenic PEDG. Treatment with LBP either completely or partially reversed the alterations caused by diabetes. It is concluded that the LBP protects retinal function and morphology in diabetic rats, probably through reinstallation of the balance between proangiogenic and antiangiogenic factors, which reduces neovascularization. LBP could be used as a therapeutic drug for DR.

## 1. Introduction

Diabetic retinopathy (DR) is one of the most common complications of diabetes mellitus (DM). It has become the most frequent cause of impaired visual acuity and blindness in working-age people in developed countries [1, 2]. DR affects more than 90% diabetic patients in clinic [3, 4]. Hitherto, it has been considered to be a microcirculatory disorder of the retina, characterized by retinal vascular leakage, inflammation, and abnormal neovascularization [5, 6]. Its consequences are breakdown of the blood-retinal barrier; retinal edema, neovascularization, and detachment; and, finally, loss of vision [7]. However, increasing evidence suggests that abnormalities of the retinal neurons and glial cells are early signs in the pathogenesis of DR [8–10]. Visual loss from DR can be reduced by partial and pan-retinal photocoagulation, laser therapy, and vitrectomy. However, these treatment procedures do not seem to significantly improve vision. Intensive glycemic control remains an important approach to prevent the development of DR [11]. It is necessary to find new compounds that could either prevent or improve DR.

Lycium barbarum polysaccharide (LBP), an extract from Lycium barbarum fruits (aka wolfberry, fructus lycii, Gouqizi, and Goji berries), is believed to be the main chemical component responsible for multiple pharmacological and biological functions of Goji berry. It is a traditional Chinese herbal medicine and has been widely utilized as a medicinal plant in China. It has been shown to enhance the immune system and to protect hepatic and nervous system [12]. As reported previously, natural products turn out to be a valuable reservoir for searching novel drugs [13]. Studies have shown that LBP possesses antidiabetic and antinephritic effects through modulating NF-*κ*B-mediated antioxidant and antiinflammatory activities [14], protects human lens epithelial cells from H_2_O_2_-induced apoptosis by reducing the generation of ROS [15], and prevents ischemia induced retinal damage by activating Nrf2 increasing HO-1 protein [16]. However, it is not known whether LBP is capable of curtailing or delaying the onset of DR. The aim of this study is to explore the effects of LBP in the retinas of diabetic rats.

## 2. Results

### 2.1. Body Weights and Blood Glucose Levels

**Figure 1:**
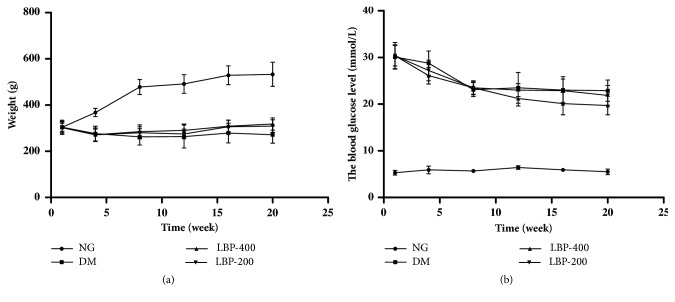
**Average weight (a) and blood glucose level (b) of control and diabetic rats. **NG, normoglycemic control group; DM, diabetic control group; LBP400, LBP 400 group; LBP200, LBP 200 group. Body weight increased during the 20 weeks experimental period in NG group, while it remained stable except an initial reduction in all diabetic groups. Blood glucose levels increased significantly in 3 diabetic groups compared with NG group.

The body weights of the rats are shown in Figure 1(a). The results showed that the body weights of the control rats increased significantly at 5 and 10 weeks after the experiments and continuously increased, but with a decreased pace, between 10 and 20 weeks. In contrast, the body weights of the diabetic rats slightly decreased at 5 weeks and maintained stable from 5 to 20 weeks, which weighted significantly less than the control animals (*P*<0.01). The body weights of the LBP200 and LBP400 animals were the same as in diabetic animals (*P*>0.05).

The blood glucose levels of experimental groups are given in Figure 1(b). The glucose contents of normoglycemic control animals were 5.3 ± 0.5 from the beginning of the experiment and fluctuated within a narrow range in the following 20 weeks. The blood glucose levels of the diabetic rats increased markedly to 30.1 ± 2.6, 7 days after streptozotocin (STZ) injection, declined slightly to 23.1 ± 1.5 after 10 weeks, and stabilized at 23.0 ± 2.9 between 10 and 20 weeks (*P*<0.01 at all observed points). The blood glucose levels of the LBP200 and LBP400 groups were identical to those of the diabetic rats (*P*<0.01* vs.* normoglycemic control;* P*>0.05* vs.* diabetic rats).

### 2.2. Effects of LBP on the Retinal Function of Diabetic Rats

**Figure 2:**
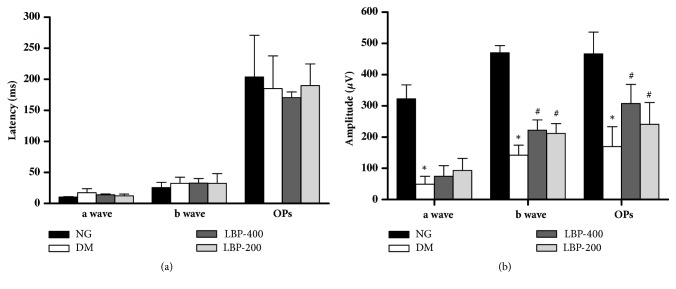
**Retinal function was assessed by ERG.** (a) Latencies of *α*-wave, *β*-wave, and OPs in different groups were not significantly changed; (b) Amplitudes of *α*-wave, *β*-wave, and OPs were suppressed by diabetes and improved by LBP treatments. *∗P*<0.05 compared with the NG group;  ^#^*P*<0.05 compared with the DM group.

To investigate the effects of LBP on the retinal function in diabetic rats, ERG analyses were performed at the end of 20 weeks. The latencies of a-wave, b-wave, and oscillatory potentials (OPs) among the four experimental groups did not significantly differ (Figure 2(a)) and LBP 200 or LBP400 did not affect the latencies of the a-wave, b-wave, and OPs. The amplitudes of the a-wave, b-wave, and oscillatory potentials (OPs) were drastically reduced in diabetic model rats compared to those of the control rats (*P* < 0.05; Figure 2(b)). Treatment with LBP200 and LBP400 moderately blunted the diabetes-induced amplitude decreases of the a-wave, b-wave, and OPs. However, the LBP was unable to completely reverse the decreases caused by diabetes, suggesting that LBP could partially improve retinal functional performance.

### 2.3. Blood Flow in the Central Retinal Veins

**Figure 3:**
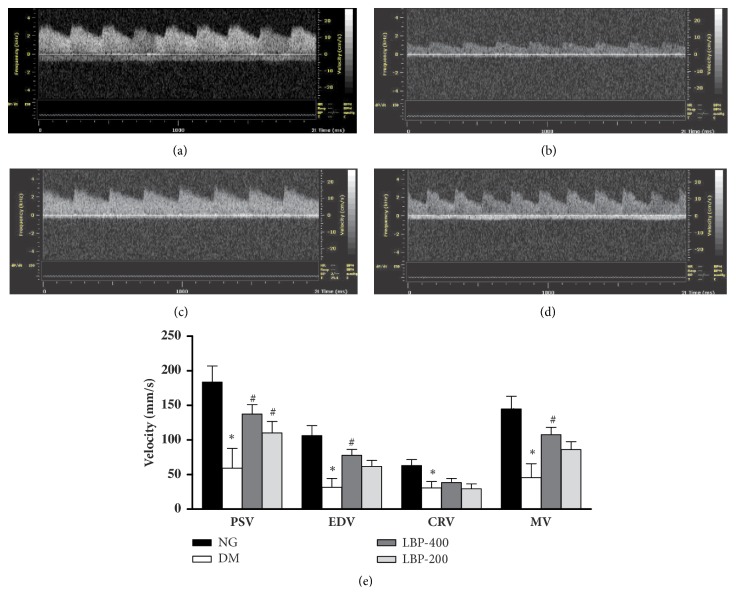
**Retinal color Doppler flow in control (a), diabetes (b), LBP-400 (c), and LBP-200 (d) groups. **(a)–(d) Representative color Doppler image showing blood flow in the central retinal artery and central retinal vein (curve below 0 in Y-axis). (e) Measurements of peak systolic velocity (PSV), end-diastolic velocity (EDV), central retinal vein velocity (CRV), and mean flow velocity (MV). Diabetes decreased PSV, EDV, CRV,and MV. LBP treatment increased these velocity measurements compared with the diabetic control group. *∗P*<0.05 compared with the control group;  ^#^*P*<0.05 compared with the model group.

The hemodynamic parameters in the four experimental groups were measured using the high resolution imaging system and shown in Figure 3(a). The mean systolic peak velocity (PSV), mean end-diastolic velocity (EDV), central retinal vein velocity (CRV), and mean velocity (=[PSV/EDV]/2) were measured and summarized in Figure 3(b). The curve above the 0 Y-axis represents central retinal artery flow and the curve below 0 Y-axis represents the central retinal vein flow. In the normoglycemic control rats, the typical waveforms from the central retinal vessels were exhibited as systolic peaks and followed by a continuous flow during diastole: the CRV below the zero axis with low and continuous flow. Compared to the normoglycemic control, the PSV, EDV, CRV, and MV were significantly decreased in diabetic control rats (*P*<0.01). Treatment with PBP 400 or LBP200 significantly improves the 4 parameters.

### 2.4. LBP Ameliorated Diabetes-Induced Retinal Structural Abnormalities

**Figure 4:**
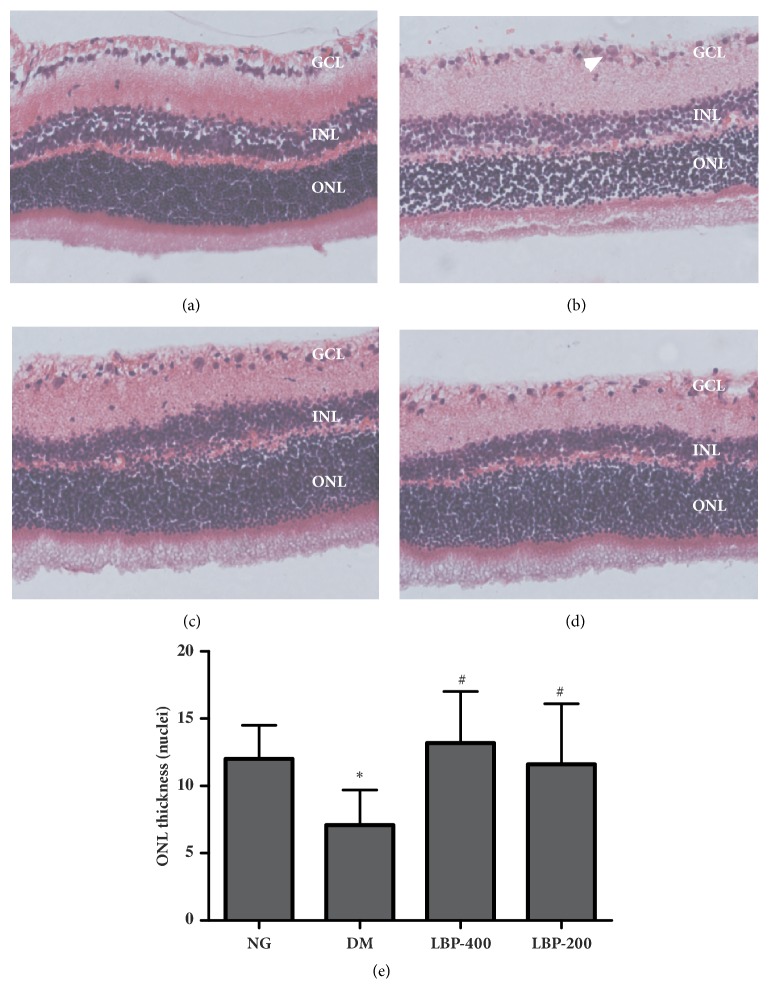
**Representative photomicrographs of hematoxylin and eosin (H&E) stained retinal sections. **In control retina, the cells in the inner nuclear layer and ganglion cell layer are uniformly distributed. In diabetic retinas (20 weeks after onset of diabetes), large number of pyknotic nuclei appear in the inner nuclear layer and there are areas of cellular dropout in the ganglion cell layer. The thinner ONL is observed in DM retina (b), while LBP preserved the thickness of the ONL ((c) and (d)). Quantification of the ONL thickness is given in (e).

In the retinas of the normoglycemic rats (Figure 4(a)), the inner and outer segments of photoreceptor and the photoreceptor nuclei were well aligned. In the retinas of the diabetic model rats (Figure 4(b)), the inner and outer segments of photoreceptor showed moderate disorganization and there were large numbers of pyknotic nuclei in the inner nuclear layers. Treatment with LBP400 (Figure 4(c) and LBP200 (Figure 4(d)) in diabetic rats improved disorganization of the inner and outer segments of photoreceptor and reduced the numbers of pyknotic nuclei. The thickness of retinas in diabetic model rats was thinner than that of the normoglycemic control rats. LBP treatment increased the thickness of retinas compared to diabetic model rats (Figures 4(c) and 4(d)). The retinas of the control rats had a densely packed ganglion cell layer (GCL) with very little space between the cells (Figure 4(a)). In contrast, retinas from diabetic model rats showed a loss cellar arrangement in the GCL, with certain areas being devoid of any cells. LBP treatment in diabetic rats improved the alterations caused by diabetes. A summarized thickness of the ONL layer is presented in Figure 4(e). As shown, the thickness of outer nuclear layer (ONL) significantly decreased in diabetic rats and LBP treatment was capable of completely reversing the changes caused by diabetes. Treatment with LBP400 in normoglycemic control animals did not alter the observed morphological indices of the retina (data not shown).

### 2.5. The Ultramicrostructure of Retinas

**Figure 5:**
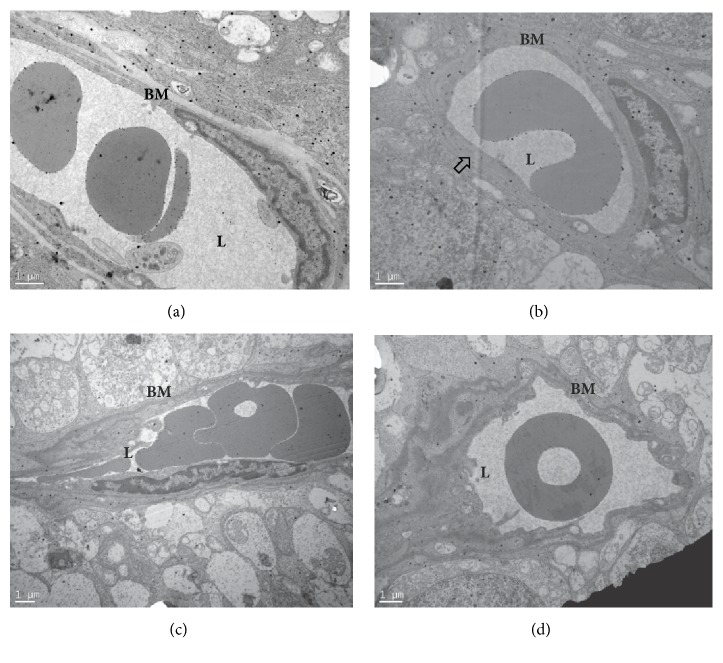
**The Ultramicrostructure of the retina of rats in control (a), diabetes (b), LBP-400(c), and LBP-200(d) groups. T**he basement membrane (BM) appeared thicker in diabetic rats than normal control. LBP-treated diabetic retinas were greatly improved and similar to those in the normal controls, n = 5/group. Magnification 5000**×.**

In the retinas of the normoglycemic control rats, the retinal capillaries had a regular structure, and the endothelial cells were close to the luminal surface. There were close continuous connections between the cells and the basement membranes (Figure 5(a)). However, in the diabetic rats, the retinal capillary endothelial cells were swollen and had more cytoplasmic pinocytosis vesicles (arrow in Figure 5(b)), and the basement membranes (BM) showed obvious thickening (Figure 5(b)). In the LBP-treated diabetic rats, the lumens of the retinal capillaries more closely resembled those of the nondiabetic rats, with reduced thickness of retinal vascular basement membrane and close attachment to the endothelial cells (Figures 5(c) and 5(d)).

### 2.6. Immunohistochemistry of GFAP, VEGF, and PEGF

**Figure 6:**
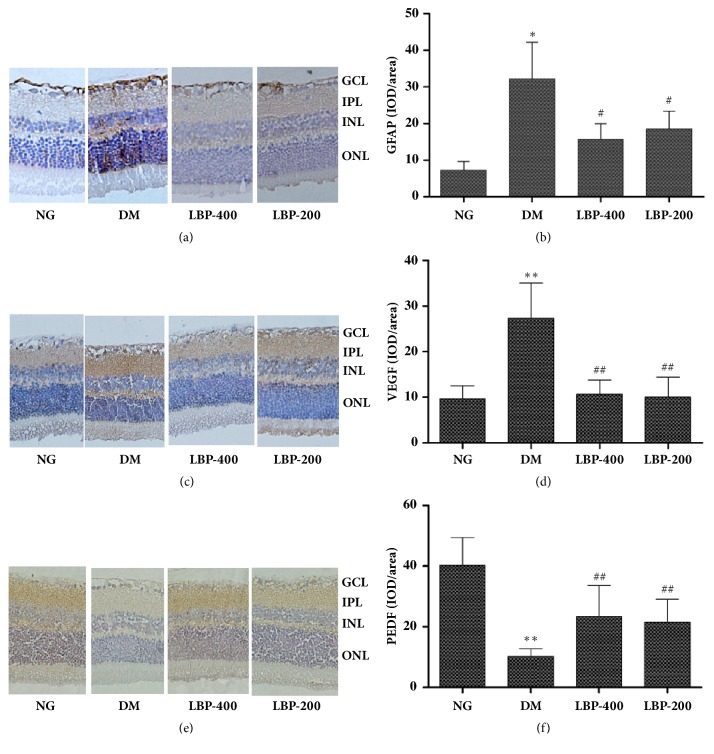
**Immunohistochemical Analysis of GFAP protein (a), VEGF (c), and PEDF (e) in the retinas of the rats. **Densitometric quantifications of immunoreactivity are in (b), (d), and (f). Diabetes significantly increased the immunoreactivities of GFAP and VEGF and decreased PEDF. LBP treatment suppressed levels of GFAP and VEGF and elevated PEDF. GCL, ganglion cell layer; IPL, inner plexiform layer; INL, inner nuclear layer; ONL, outer nuclear layer. *∗P*< 0.05 compared with control group; #*P*< 0.05, compared with model group.

Glial fibrillary acid protein (GFAP) immunohistochemistry revealed faint staining in the nerve fiber layer and retinal GCL of the retinas in normoglycemic control rats (Figure 6(a)). The GFAP staining was significantly enhanced throughout the whole vertical section of the diabetic rat retinas, especially in the Müller cells (Figure 6(a)) in the diabetic model rats. Measurement of GFAP immune-intensity showed a significantly higher level in diabetic rats than the control rats (*P*<0.05, Figure 6(b)). Treatment with LBP400 and LBP200 significantly decreased the immune-intensity of GFAP compared with diabetic control rats (*P*<0.05, Figure 6(b)).

Vascular endothelial growth factor (VEGF) staining showed virtually no VEGF-positive labeling in the normoglycemic control rats (Figure 6(c)). VEGF-positive labeling was apparent in the retinas of diabetic model rats, especially in GCL and ONL layers (Figure 6(c)). LBP400 and LBP200 reduced the intensity of the VEGF staining. Semiquantification of VEGF immune-intensity demonstrated that VEGF staining was significantly increased in the diabetic rats compared with the normoglycemic control (*P*<0.01, Figure 6(d)) and that LBP treatments reversed the diabetes-induced VEGF elevation (*P*<0.01, Figure 6(d)).

Pigment epithelium derived factor (PEDF) staining results were opposite to those of the GFAP and VEGF. The PEDF positive staining was observed in IPL of the retinas in the normoglycemic control animals (Figure 6(e)). It was significantly suppressed by diabetes and partially reversed by LBP treatment. The immune-intensity measurements revealed that the PEDF levels were significantly lower in diabetic than in normoglycemic control rats (*P*<0.01) and that intervention with LBP400 and LBP200 partially ameliorated the diabetes-induced decline of PEDF (*P*<0.01 vs. diabetic control, Figure 6(f)).

### 2.7. Western Blot Analyses of GFAP, VEGF, and PEDF

**Figure 7:**
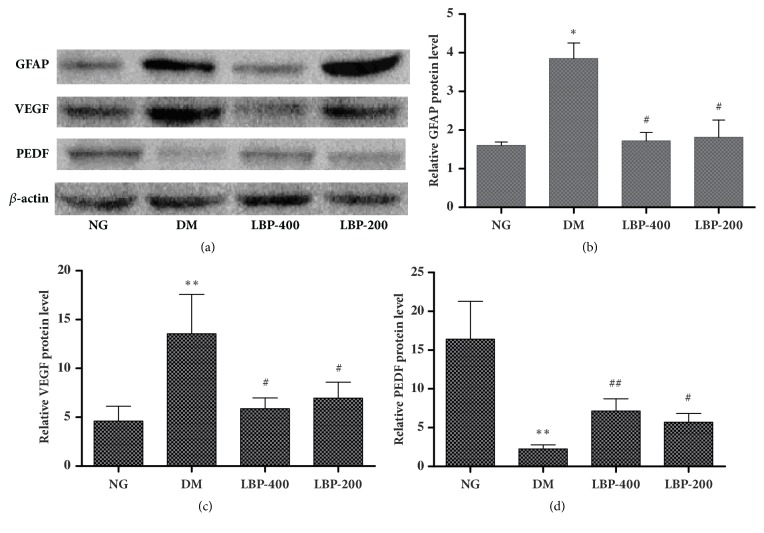
**The effect of LBP on the expression of GFAP, VEGF, and PEDF in the retinal tissue. **(a) Representative western blots. (b)–(d) Relative quantification of GFAP, VEGF, and PEDF. Diabetes increased protein levels of GFAP and VEGF and decreased PEDF. LBP completely reversed GFAP and VEGF and partially reversed PEDF levels. *∗P*<0.05, *∗∗P*<0.01, compared with the NG group; ^#^ * P*<0.05, ^#^  ^#^ * P*<0.01, compared with the DM group.

The representative western blots of GFAP, VEGF, and PEDF are shown in Figure 7(a) and semiquantitative band densitometry measurement results are given in Figures 7(b)–7(d). The results were consistent to those observed in immunohistochemistry. Thus, both GFAP and VEGF contents were significantly higher in the retinas of the diabetic model rats compared with the control rats (*P* < 0.05,* P* < 0.01, respectively; Figures 7(b) and 7(c)). Treatment with LBP400 and LBP200 returned the levels back to normoglycemic control level. On the contrary, PEDF protein level was significantly decreased in diabetic compared with normoglycemic control rats (*P*<0.01, Figure 7(d)). LBP400 significantly (*P*<0.01) and LBP200 moderately (*P*<0.05) increased the levels of PEDF; however, the increases only reach to about 40% of the level in the normoglycemic control rat.

### 2.8. mRNA Expressions of GFAP, VEGF, and PEDF

Expressions of the GFAP, VEGF, and PEDF genes were detected using quantitative real-time PCR. The results were consistent to those observed by immunohistochemistry and western blotting. Therefore, modest quantities of GFAP and VEGF mRNA were expressed in the normoglycemic control rats and significantly increased in the diabetic control rat (*P*<0.05 and* P*<0.01, respectively; Figures 7(a) and 7(b)). Treatment with LBP reduced the GFAP mRNA levels to the control baseline and suppressed the diabetes-caused increase of the VEGF. The effects of LBP400 were slightly better than LBP200; however such mild difference did not reach to a statistical significance.

**Figure 8:**
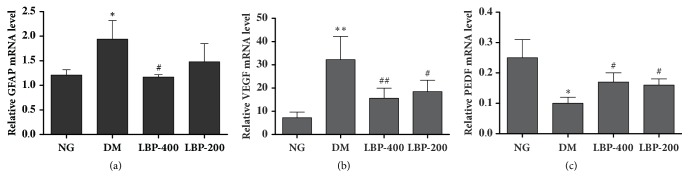
**The effect of LBP on mRNA expression of GFAP, VEGF, and PEDF in the retinal tissue. **Diabetes increased mRNA expression of GFAP and VEGF, whereas it suppressed PEDF gene expression. LBP ameliorated the diabetes-induced alterations. *∗P*<0.05,*∗∗P*<0.01, compared with the NG group; ^#^ * P*<0.05, ^#^  ^#^ * P*<0.01, compared with the DM group.

The change of PEDF is opposite to that observed in GFAP and VEGF. The PEDF mRNA expression level was relatively high in the normoglycemic controls and it was significantly suppressed by diabetes (*P*<0.05, Figure 8(c)). Treatment with LBP partially elevated the levels of PEDF (*P*<005).

## 3. Discussion

Diabetic retinopathy (DR) is one of the most common microvascular complications of diabetes. A study reported that about one-third of the diabetic patients have certain signs of DR and about one-tenth of patients have vision-threatening retinopathy [17]. Although a lot of important information or clues on the development of DR have obtained from human studies, the mechanisms of DR development still elusive. The Type I Diabetes (TID) rat model has been widely used by visual scientists to analyze molecular mechanisms associated with diabetic retinopathy [18]. In the present study, the TID rat model was successfully established by STZ injection. The animals manifested the characteristic of hyperglycemia and impaired retinal function.

Lycium barbarum berries have been used in traditional Chinese medicine for thousands of years. The berries contain abundant polysaccharides, scopoletin, carotenoids, betaine, cerebroside, beta-sitosterol, flavonoids, amino acids, minerals, and vitamins (particularly riboflavin, thiamin, and ascorbic acid) [for review, please see [19]]. It is believed, however, that the water-soluble LBPs are the major components in the berries that possess a wide array of pharmacological activities. LBPs contain a mixture of partially characterized polysaccharides and proteoglycans [20, 21]. Different fractions of LBP possess different functions. In general, LBPs have been shown to possess antitumor [22], immune-regulatory [23], hepatoprotective, and neuroprotective properties [24]. Recently, it has been reported that LBP protects human lens epithelial cells and retina after ischemia-reperfusion [25] and improves obesity and diabetic complications in cells and animals [26]. In this study, we have employed a water-soluble LBPs mixture for animal study.

The present study is the first report to our knowledge showing that LBP improves diabetes-induced retinal alterations at both the functional and structural levels. LBP ameliorated diabetes-induced abnormalities in nerve electrophysiology (ERG), hemodynamic measurements (Figures 2-3), and anatomical structures (Figures 4-5). Our study further revealed that activation of GFAP and VEGF and suppression of PEDF may be the underlying mechanisms that diabetes causes retinal alterations. Finally, we demonstrated that LBP intervention ameliorated diabetes-caused retinal changes partially or completely.

In the present study, in order to confirm the effects of LBP on functional changes in diabetic retinopathy, we analyzed retinal function using ERG and color Doppler image. The amplitudes of the a-wave, b-wave, and OPs in ERG were reversed by application of LBP in diabetic rats (Figure 2). Ultrasound is a classic diagnostic tool retinopathy [27–29]. It has the ability to obtain quantitative measurements of vascular flow. The hemodynamic parameters including PSV, EDV, CRV, and MV were measured. While diabetes resulted in significant decreases of PSV and EDV in the central retinal artery, LBP treatments reduced the changes caused by diabetes. These results suggest that LBP-mediated is capable of ameliorating retinal dysfunction in diabetic animals.

Retinal cell death causes reduction in thickness of various layers of the retina, which leads to overall thinning of the retina. These changes have been reported in diabetic retinopathy in both experimental animals and clinical patients using optical coherence tomography [30, 31]. In the present study, we observed decreased thickness of retinal layers in diabetic retina. Treatment with LBP in diabetic rats almost completely reversed the diabetes-induced reduction. Moreover, ultramicrostructural study using electron microscope revealed thickening of blood vessel wall and reduced lumen diameter in the retina of diabetic rat. LBP treatments, however, reduced the thickness of the basement membrane and increased the vessel lumen size.

To explore the mechanisms underlying the protective effects of LBP on diabetic retinopathy, we measured the mRNA and protein levels of GFAP, VEGF, and PEDF using immunohistochemistry, western blotting, and quantitative real-time PCR. GFAP is an established indicator of retinal stress. In the normal mammalian retina, GFAP is marginally detectable in Müller cells, which are the principal glial cells in vertebrate retina that regulate the function of retinal neurons and maintain the integrality of blood-retinal barrier. Once being stressed, activated Müller cells express high levels of GFAP. In the present study, increased GFAP expression was observed in Müller cells, indicating that Müller cell dysfunction was involved in STZ-induced diabetic retinopathy, which is consistent with previous studies [32, 33]. Neuronal dysfunction or cell loss in diabetic retinas might partly be due to Müller cell dysfunction. Our study confirmed that GFAP are markedly upregulated in diabetic retinas, especially in Müller cells, as compared to normal retina. LBP treatment ameliorated high glucose-induced Müller cell dysfunction and GFAP overexpression.

The retina is a cardinal layer for vision as it transforms the incoming light into neural activity. This conversion requires energy and thus an adequate supply of nutrients by blood vessels to the retina is critical [34, 35]. An overly rich vascular network on the inner side of the retina would interfere with the transmission of light through the retina. So the retina must have a fine tuned balance between a sufficient blood supply to the retina and a minimal interference with the light path to the photoreceptors [36]. Pathological retinal neovascularization is a feature of diabetic retinopathy and other ocular disorders characterized by retinal hypoxia [37]. As in other tissues, retinal angiogenesis depends on the balance between proangiogenic and antiangiogenic factors. VEGF and PEDF act as important factors in regulating vascular leakage and forming new blood vessels. VEGF is the main blood vessel stimulating factor and PEDF is the angiogenesis inhibitory factor. The imbalance of VEGF and PEDF can cause vascular damage, disruption, angiogenesis, and neovascularization. The newly formed blood vessels are fragile and prone to hemorrhage, which can impair vision, ultimately causing blindness. Indeed, an inverse correlation between vitreous PEDF and VEGF levels in patients with diabetic retinopathy has been described [38]. VEGF controls several processes, such as proliferation, survival, and migration of blood vessel endothelial cells. Retinal VEGF expression is correlated with diabetic blood-retinal barrier breakdown and neovascularization in animals and humans [39, 40]. In the present study, VEGF expression was significantly upregulated in diabetic retina, indicating that VEGF overexpression plays a crucial role in retinal vascular abnormality in STZ-induced diabetes. In the eye, PEDF expression is regulated in an opposite manner to VEGF. PEDF possesses powerful antiangiogenic properties, especially to newly formed vessels, which can effectively inhibit the development of neovascularization in diabetic rat [41, 42]. Our results also suggested that PEDF expression was decreased in diabetic retina. However, LBP treatments inhibited VEGF overexpression and increased PEDF expression. These data suggest diabetes leads to imbalance between VEGF and PEDF and LBP restores the balance between the two.

In conclusion, the present study demonstrates that diabetes impairs retinal functional performances and alters the ultrastructure of the retinal cells. Diabetes-induced retinopathy may be associated with upregulation of proangiogenic GFAP and VEGF and suppression of antiangiogenic PEDF. LBP treatment in diabetic rats improves the retinal functional performance and ameliorated structural changes. These effects are associated with restoration of the balance among GFAP, VEGF, and PEDF. Our present data provide molecular evidence of the potential validity of LBP supplementation as a therapeutic strategy to prevent retinal degeneration related to diabetic retinopathy. Further study to reveal the mechanisms of LBP action is warranted.

## 4. Materials and Methods

### 4.1. Diabetic Animal Models and Drug Administration

Male Sprague-Dawley rats weighed 250 ± 20 g were obtained from the Ningxia Medical University Experimental Animal Center (certificate ID NXSY2011-0001A). All animals were housed at room temperature (22~25°C) and 45% humidity, with 12-hour light/dark cycles, and free access to food and water. All animal procedures followed the NIH Guide for the Care and Used of Laboratory Animals and the protocol was approved by the Institutional Animal Care and Use Committee at Ningxia Medical University.

After adaptive feeding for one week, rats were starved for 12 hours before being given an intraperitoneal injection of streptozotocin (STZ) (45 mg/kg; 0.45% STZ solution with 0.1 mmol/L citrate buffer, pH 4.5). To protect the rats from the otherwise fatal hypoglycemic effects of pancreatic insulin release, 10% glucose solution was provided 6 hours after STZ injection and lasted for the next 24 h. Citrate buffer injected rats served as controls. The blood glucose levels of the rats were measured from their tail tips using a glucometer (FreeStyle Freedom, Abbott, America) one week after the STZ injection. Rats with fasting blood glucose levels above 16.7 mmol/L were classified as diabetic rats. The diabetic rats were divided into four groups, namely, normoglycemic control, diabetic control, LBP400 (LBP 400 mg/kg/d), and LBP200 (LBP 200 mg/kg/d) groups. The selection of the LBP doses was based on previously publication showing that LBP 200 and 400 mg/kg provided potent protection to retinal cells against N-Methyl-N-Nitrosourea induced apoptosis in animals [43]. LBP was given orally for the next 20 weeks. Fifteen normal Sprague-Dawley rats, with matched body weight, age, and gender, were served as controls. Body weights and fasting blood glucose levels were recorded every fourth week.

### 4.2. Electroretinogram (ERG) Recordings

Rats were adapted to the dark environment for at least 70 minutes prior to ERG recording. In brief, the rats were anesthetized with ketamine (70 mg/kg body weight) and xylazine hydrochloride (10 mg/kg body weight) injection intraperitoneally. Pupils of rats were then dilated using 0.5% tropicamide. ERGs of both eyes were recorded from the corneal surface using a silver chloride electrode loop encased in a layer of 1% methylcellulose. Two reference electrodes were placed in the subcutaneous tissue behind the ears and a ground electrode was placed in the subcutaneous tissue of the tail. Full-field (Ganzfield) stimulation was applied, and amplitudes and latencies of a-wave and b-wave as well as OPs were recorded using a Roland Consult Electrophysiological Diagnostic System (Brandenburg, Germany).

### 4.3. Blood Flow Measurements from the Central Retinal Blood Vessels

The Visual Sonics Vevo 770™ system (VisualSonics, Canada) was used for the ultrasound measurement of retinal blood flow velocity parameters. Rats were anesthetized with 2~3% isoflurane in O_2_ and positioned on the heated table of the ultrasound machine and then the isoflurane was reduced to 1~1.5% isoflurane during the blood flow measurement. The ultrasound transducer, coupled with the ultrasound gel, was applied to the surface of the eye, and a RMV-710B probe was used for PW Doppler Mode image acquisition. The central retinal artery locates in the region of the optic nerve, approximately 16 mm behind the globe. A high resolution imaging system with a 25 MHz transducer was used to measure retinal blood flow velocities. PSV, EDV, and CRV were recorded. MV were calculated with formula MV = (PSV+EDV)/2.

### 4.4. Processing of Retinal Tissue

All animals were sacrificed at the end of week 20 by intraperitoneal injection of 1% pentobarbital sodium (50 mg/kg). The right eyes were removed and fixed in 4% paraformaldehyde solution for histological studies. The left eyes were removed and incised along the limbus of the cornea to remove the cornea, lens, and vitreous humor. Ophthalmic knives were used to remove the retinas, which were then immediately frozen in liquid nitrogen, and stored at –80°C for subsequent biochemical analysis.

### 4.5. Retinal Examination by Optical Microscopy

Ocular bulbs were immersed in 4% paraformaldehyde fixative and then embedded in paraffin, from which 5 mm thicknesses slices were cut. Ten micron thick cryosections were obtained (Shandon AS325 Retraction) and stained with hematoxylin and eosin (H & E) for microscopic examination using an Eclipse Nikon E800 Microscope (Tokyo, Japan). A minimum of five sections per eye were examined.

### 4.6. Retinal Examination by Electron Microscopy

Ocular bulbs were prefixed with 2.5% glutaraldehyde solution and postfixed with 1% osmium tetroxide solution, followed by a gradient dehydration with ethanol and acetone. They were then saturated, embedded, polymerized, and solidified with pure epoxy resin, Epon812. Ultrathin sections were cut, stained with uranyl acetate for 30 minutes, and washed for 3 minutes with distilled water. The moisture was removed with filter papers, naturally dried, and stained with lead citrate for 30 minutes. The sections were washed again for 3 minutes with distilled water, the moisture was absorbed with filter papers, and then they were naturally dried.

### 4.7. Immunohistochemistry Analysis

The ocular bulbs were fixed for 48 hours in 4% paraformaldehyde (Sigma-Aldrich, St Louis, MO), embedded in paraffin, and then sectioned. The sections were dewaxed using xylene, dehydrated through a graded ethanol series, and oxidized using a 3% H_2_O_2_ solution for 15 minutes at room temperature. Polyclonal mouse anti-GFAP antibody (1:2000, Cat#ab53554, Abcam, Cambridge, UK), polyclonal rabbit anti-VEGF antibody (1:100, Cat#ab184784, Abcam, Cambridge, UK), and polyclonal rabbit anti-PEDF antibody (1:200, Cat#ab180711, Abcam, Cambridge, UK) were added and incubated at 4°C overnight in a humid chamber. Then, after washing with phosphate buffered saline (PBS) the next day, a secondary antibody was applied for 2 hours. PBS was substituted for the first antibody, as a negative control. Brown staining of tissue and blue staining of nuclei were taken as positives for detection of the relevant antigens. To quantify the expression of the antigens, images were acquired using an optical microscope (magnification × 400) and six random fields in each section were analyzed. Integral optical density values of each visual field were measured using Image Pro PLUS 6. 0 software.

### 4.8. Western Blot Analysis

Retinal tissues were homogenized on ice using 400 *μ*l of RIPA Lysis Buffer containing phenylmethanesulfonyl fluoride (PMSF). The homogenates were then centrifuged at 12000 rpm at 4°C for 5 minutes. The concentrations of extracted proteins in the supernatant were determined using a protein assay kit (BCA, Pierce Biotechnology), according to the manufacturer's instructions. Samples containing 50 *μ*g of protein were separated using SDS sodium dodecyl sulfate-PAGE (polyacrylamide gel electrophoresis), then electrophoresed, and transferred to polyvinylidene fluoride (PVDF) membranes. After blocking with 5% skimmed milk, the membranes were incubated with polyclonal mouse anti-GFAP antibody (1:10000), polyclonal rabbit anti-VEGF antibody (1:1000), polyclonal rabbit anti-PEDF antibody (1:1000), or rabbit anti-*β*-actin (1:5000, bs-0061R, BIOSS, CHINA) at 4°C overnight. The membranes were washed and then incubated with horseradish peroxidase-conjugated goat anti-rabbit IgG (1:5000) secondary antibody, at room temperature for 1 hour. Enhanced chemiluminescence was used for detection. A Bio-Rad image analysis system was used to scan the optical density of the target bands, and Quantity One software was used to analyze the relative optical density of GFAP, VEGF, PEDF, and *β*-actin.

### 4.9. Real-Time Quantitative PCR

The total RNA was isolated from the retinas with TRIZOL extraction kit (Invitrogen), according to the manufacturer's protocol. The quality and quantity of the RNA prepared from each sample were determined by ultraviolet (UV) absorbance spectroscopy. cDNA was made by reverse transcription, using a RevertAid™ First Strand cDNA Synthesis Kit. Retina RNA (2 *μ*g) was converted into cDNA in a total reaction volume of 25 *μ*l, containing 1 mg Oligo (dT), 5 *μ*l M-MLV 5 × buffer, and 1.25 *μ*l dNTP. The mixture was incubated for 60 minutes at 42°C and the reaction stopped by heating at 95°C for 5 minutes. The target gene primers used for the detection of GFAP, VEGF, PEDF, and *β*-actin were as follows:

  GFAP (F) 5′-TCTGCCCAGTGAGTAAAGGTGA-3′
  GFAP (R) 5′-GGTGTGGAGTGCCTTCGTATTA-3′
  VEGF (F) 5′-TAG ACC TCT CAC CGG AAA GAC-3′
  VEGF (R) 5′-CAG GAA TCC CAG AAA CAA AAC-3′
  PEDF (F) 5′-GACTATCACCTTAACCGACC-3′
  PEDF (R) 5′-TTTTATTGCAGAGGCTACAT-3′
 
*β*-actin (F) 5′-ATC ATG TTT GAG ACC TTC AAC-3′
 
*β*-actin (R) 5′-CAT CTC TTG CTC GAA GTC CAA-3′

The reactions were executed using the following system: 2 × SYBR Green qPCR mix (modified DNA polymerase, SYBR Green I, Optimized PCR buffer, 5 mmol/L MgCl_2_, dNTP mix) 12 *μ*l, forward primer 1 *μ*l, reverse primer 1 *μ*l, cDNA template 2 *μ*l, made up with water to 25 *μ*l. The PCR amplification reaction conditions were 94°C for 2 minutes, 94°C for 30 seconds, 50°C for 30 seconds, and 72°C for 30 seconds (35 cycles). Each run was repeated three times, along with three nontemplate negative controls. Melting curve analysis was used to ensure the purity of the amplified PCR product. Fluorescence was measured throughout the process. After the reaction, the Ct values of the samples were calculated at the point at which they reached the threshold value during the process of PCR amplification, and the relative mRNA levels of GFAP, VEGF, and PEDF in each sample were normalized to *β*-actin expression.

### 4.10. Statistical Analysis

Statistical analysis was performed using SPSS software package (SPSS for Windows, version 18.0, USA). Results were expressed as means ± standard deviations (SDs). Differences were assessed using one-way analysis of variance (ANOVA). A level of* P* < 0.05 was considered statistically significant. The Tukey post hoc test was used to determine differences between groups. Each subgroup contains at least 3 samples.

## Data Availability

Conclusions in this article are based on statistical analysis using the averages and standard deviations obtained from multiple experimental trials as indicated in the methods. Figures in this article that present images from western blots, histology sections, and Doppler flow are representative from these trials. To obtain additional images or raw data used to calculate averages presented in this article, the corresponding author can be contacted at yaoqing726@163.com.

## Abbreviations

DR: Diabetic retinopathy
DM: Diabetes mellitus
TID: The Type I Diabetes
LBP: Lycium Barbarum Polysaccharide
ERGs: Electroretinograms
PSV: The mean systolic peak velocity
EDV: Mean end-diastolic velocity
CRV: Central retinal vein velocity
MV: Mean velocity
GFAP: Glial fibrillary acid protein
VEGF: Vascular endothelial growth factor
PEDF: Pigment epithelium derived factor
GCL: Ganglion cell layer
ONL: Outer nuclear layer.
